# Molecular Mechanisms of Survival Strategies in Extreme Conditions

**DOI:** 10.3390/life2040364

**Published:** 2012-12-07

**Authors:** Salvatore Magazù, Federica Migliardo, Miguel A. Gonzalez, Claudia Mondelli, Stewart F. Parker, Beata G. Vertessy

**Affiliations:** 1 Department of Physics, University of Messina, Viale D’Alcontres 31, P.O. Box 55-98166, Messina, Italy; E-Mail: fmigliardo@unime.it; 2 Institut Laue Langevin, 6, Rue Jules Horowitz, F-38042 Grenoble Cedex 9, France; E-Mail: gonzalez@ill.fr; 3 CNR-IOM-OGG, Institut Laue Langevin, 6, Rue Jules Horowitz, F-38042 Grenoble Cedex 9, France; E-Mail: mondelli@ill.fr; 4 ISIS Facility, Rutherford Appleton Laboratory, Chilton, Oxon, OX11 0QX, UK; E-Mail: stewart.parker@stfc.ac.uk; 5 Institute of Enzymology, Research Center for Natural Sciences, Hungarian Academy of Science, Budapest, Hungary; E-Mail: vertessy@enzim.hu (B.G.V.); 6 Department of Applied Biotechnology and Food Sciences, University of Technology and Economics, Budapest, Hungary

**Keywords:** survival strategies, molecular mechanisms, lysozyme, dUTPase, trehalose, structure, dynamics, spectroscopic techniques

## Abstract

Today, one of the major challenges in biophysics is to disclose the molecular mechanisms underlying biological processes. In such a frame, the understanding of the survival strategies in extreme conditions received a lot of attention both from the scientific and applicative points of view. Since nature provides precious suggestions to be applied for improving the quality of life, extremophiles are considered as useful model-systems. The main goal of this review is to present an overview of some systems, with a particular emphasis on trehalose playing a key role in several extremophile organisms. The attention is focused on the relation among the structural and dynamic properties of biomolecules and bioprotective mechanisms, as investigated by complementary spectroscopic techniques at low- and high-temperature values.

## 1. Introduction

Extreme environments are widely spread on Earth, encompassing very different regions at every altitude and latitude, such as deserts, volcanoes, seafloors and mountains; analogously, very different forms of life grow and evolve by refining a wide range of survival strategies depending on stress factors, such as temperature, pressure and pH [1]. As a consequence, extremophiles, organisms living in extreme environments, are classified, for example, as hyperthermophiles and thermophiles (very high temperature), psychrophiles (very low temperature) and halophiles (high salt concentrations) [1]. 

Several extremophiles belonging to different natural kingdoms share analogous strategies to survive under various stress conditions. In this review, the attention will be focused on the study by complementary spectroscopic techniques of some bioprotectant systems, such as a disaccharide, trehalose and an alcohol, glycerol playing a key role under thermal and anhydrobiotic stresses, and on their effects on some proteins, such as lysozyme and dUTPase, found also in extremophiles. 

Some strains of *Thermus thermophilus* [2,3,4] are commonly found in marine hot springs. They grow in media containing 3% to 6% NaCl, and they produce trehalose during salt-induced osmotic stress. In the thermophilic archaeon *Sulfolobus acidocaldarius* [5,6], the pathway for the synthesis of trehalose converts the terminal unit of a glucose polymer to trehalose via maltooligosyltrehalose synthase and maltooligosyltrehalose trehalohydrolase. 

In very high saline environments, halophile organisms, such as tardigrades, nematodes and the crustacean *Artemia salina*, can tolerate extreme desiccation by passing into anhydrobiosis, a state characterized by little intracellular water and no metabolic activity. In tardigrades [7,8], a breakdown of lipid and glycogen in the cavity cells and a concomitant increase in intracellular concentrations of trehalose and glycerol occurs in anhydrobiotic conditions. Furthermore, in the nematode *Aphelenchus* [9], trehalose is accumulated during desiccation in 97% relative humidity, while glycerol amounts are found after this phase. The two bioprotectant systems therefore allow the nematode to maintain its metabolic functions even when dehydration occurs.

Furthermore dry cysts of *Artemia salina* [10,11,12,13], a crustacean known as the “brine shrimp”, are very resistant to extreme temperatures and, in anhydrobiosis, stop trehalose-based energy metabolism. The trehalose utilization and glycogen synthesis that occur during development of fully hydrated cysts are both blocked during desiccation. 

Other examples of the accumulation and interplay of trehalose and glycerol have been demonstrated to occur in desiccation and freezing conditions, as in the arctic insect *Megaphorura arctica,* where the natural synthesis of trehalose and glycerol is related to the changes in membrane composition and to the prevention of damage from dehydration [14,15].

The biological relevance of the combined trehalose and glycerol bioprotectant effect on several organisms living in anhydrobiotic and cryobiotic conditions have promoted both experimental and simulation studies [16,17,18,19,20,21,22].The cofactors making the combination of trehalose and glycerol so effective in the protein protection under stress conditions has been determined by focusing on the molecular interactions between the two systems. Trehalose and glycerol have been demonstrated to create an environment around proteins that is able to improve their thermal stability and to control their dynamics on the pico- and nano-second timescale. In such a way, the two bioprotectant systems are capable to modulate both the extent of the protein atomic mean square displacements and the onset of the dynamical transition [16,17]. A non-Debye relaxation dynamic, as a result of the combination of the effects of confinement and mixing of the two constituents, has been revealed, as well as an increase of the non-exponential character of the structural relaxation [17,18]. Furthermore, enzymes embedded in mixtures of glycerol and trehalose with various compositions showed longer deactivation times and smaller mean square displacements [19,20,21]. Finally, the antiplasticizing effect of glycerol on trehalose has been probed by dielectric studies [22].

Several proteins have shown an extraordinary capability to adapt their conformations and motions to exert their biological functions even under stress conditions. Among them, lysozyme is a well known protein that has been extensively studied by theoretical, experimental and simulation methods [23,24,25,26] because of its properties that make it a model protein to study more complex biomolecules as those found in extremophiles. It has been also pointed out that in lysozme water solutions, ordered and disordered hydration sites extended over the protein surface, suggesting the presence of a dynamic hydration layer with ionic “flip-flop” occurring between bound waters [24,25,26]. Small Angle Neutron Scattering (SANS) measurements revealed that the average interparticle distance increases in lysozyme unsaturated solutions, due to the increased interaction between molecules, progressed as the salt concentration decreased, while in supersaturated solutions, crystallization processes are activated [26]. 

Furthermore, lysozyme is responsible for breaking down the polysaccharide walls of many kinds of bacteria, so providing some protection against infection, and it is also a cold-adapted protein. It has been shown that lysozyme from the insect *Manduca sexta* possesses a higher content of α-helix secondary structure compared to that of hen egg white lysozyme. In addition, the *M. sexta* lysozyme enzymatic activity is higher, in the range of 5 °C−30 °C [27]. 

One of the few protein factors essential in both the maintenance of stable genetic information and the strict control of the nucleotide pools is dUTPase. dUTPase has been also isolated by hyperthermophilic archaeon *Thermococcus onnurineus* NA1 and in the archaeon *Pyrococcus furiosus*. In *P. furiosus*, a thermostable enzyme has been found to be a multimer of two discrete proteins, P45 and P50, the first one converting dUTP to dUMP and inorganic pyrophosphate. Archaeal dUTPases may play an essential role in preventing dUTP incorporation and inhibition of DNA synthesis by family B DNA polymerases [28].

The present review shows a plethora of spectroscopic data collected on the binary systems trehalose/water mixtures and trehalose/glycerol mixtures, as well as on the ternary systems trehalose/lysozyme/water and trehalose/dUTPase/water in order to elucidate the molecular mechanisms allowing extremophiles to survive under stress conditions.

## 2. Bioprotection Mechanisms and Extreme Conditions

### 2.1. Cryobiotic and Cryptobiotic Effects of Trehalose

From the molecular point of view, the manifold aspects of the bioprotective function of trehalose, which can explain its ubiquity, have been investigated in deep detail by using complementary spectroscopic techniques covering very wide space and time ranges [29,30,31,32,33,34,35,36,37,38]. The whole body of the collected data pointed out the fundamental role played by the interaction of trehalose with water. 

**Figure 1:**
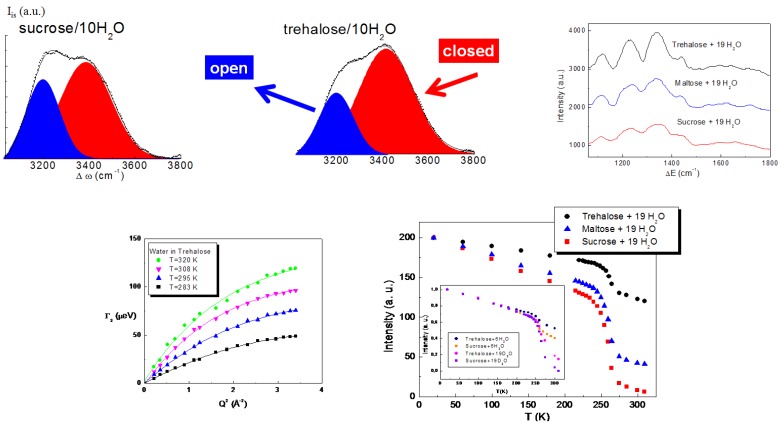
Structural and dynamic properties of trehalose/water mixtures [29,30,31,32,35,36,37,38,32,35].

The study of the structural properties of trehalose water mixtures highlighted that water molecules are arranged in the presence of trehalose in a particular configuration, which avoids ice formation, so preserving biomolecules from damage due to freezing and cooling. Neutron diffraction, Raman spectroscopy and Inelastic Neutron Scattering findings [29,30,31,32], shown in Figure 1, revealed that the addition of trehalose, with respect to the other disaccharides, completely destroys the tetrahedral intermolecular network of water, which by lowering the temperature would give rise to ice. In the vibrational spectrum of liquid water, one can distinguish the existence of an isosbestic point in the isotropic spectrum of pure water allowing the decomposition of each spectrum into an “open” contribution, attributed to the O–H vibration in tetrabonded H_2_O molecules that have an “intact bond”, and a “closed” contribution, corresponding to the O–H vibration of H_2_O molecules that have a not fully developed hydrogen bond (distorted bond). One can observe that for the same concentration, the integrated area of the “open” band is smaller in the trehalose aqueous solution. This allows us to state that a more marked destructuring effect occurs in the presence of trehalose, rather than in the presence of sucrose or maltose [29,30,32]. As a confirmation, neutron diffraction results confirm the changes induced by disaccharides on water tetrahedral structure. In fact, the peak at 4.5 Å in g_OO_(r) of pure water, which is associated with the “degree of tetrahedrality”, in the distribution function of trehalose/H_2_O mixture at a concentration value corresponding to 40 H_2_O molecules for each trehalose molecule at T = 300 K is absent, and the general trend is significantly distorted [20]. Uchida and coworkers [33] detecting freeze-fractured replica images of disaccharide (trehalose, sucrose and maltose) solutions using a field-emission type transmission electron microscope, confirmed that trehalose molecules have a greater inhibitory effect of sucrose on the growth of ice crystals, while Furuki [34] observed that aqueous trehalose has a larger amount of unfrozen water content in comparison with the other disaccharide mixtures and interpreted their different degree of anti-freeze effects in view of the molecular structure of the disaccharides, concluding that the aqueous unfrozen behavior induced by the presence of trehalose depends on the position of the glycosidic linkage between the two constituent units.

On the other hand, the study of the dynamical properties of trehalose water mixtures (Figure 1) has shown that the diffusion of water is strongly affected by trehalose and that trehalose and water form a unique entity, creating a rigid environment where biomolecules can be protected [35,36,37,38]. More specifically, Quasi Elastic Neutron Scattering results revealed that the diffusion coefficient of water in the presence of trehalose is similar to that of pure water at lower temperature, so showing that trehalose, besides imposing an order on the tetrahedral hydrogen bond network of water, significantly slows the dynamics of water. The higher slowing down effect of the diffusive dynamics observed for trehalose is evidently linked to its extraordinary capability to “switch off” metabolic functions [36,37]. Furthermore, the elastic intensity and the mean square displacement behaviors of trehalose water mixtures as a function of temperature revealed that a higher onset temperature value for trehalose, as compared to the other disaccharides together with a lower fragility of trehalose water mixtures [35,38]. It is possible to conclude that the trehalose-water system is more “rigid” than maltose-water and sucrose-water systems. From this analysis [29,30,31,32,33,34,35,36,37,38], it clearly emerges that trehalose and its water mixtures are characterized with respect to the other disaccharides and their mixtures by a superior structural resistance to thermal stress, which allows them to create a more rigid shell to protect biological structures.

### 2.2. Increased Bioprotective Effectiveness of Trehalose/Glycerol Mixtures

With the aim to investigate the different bioprotective effectiveness of trehalose and trehalose/glycerol (T/G) mixtures, a systematic study on mixtures at different glycerol concentration values in trehalose (0%, 1.25%, 2.5%, 5%, 7.5% and 10% by weight) has been performed. The goal of these experiments was to investigate the vibrational, relaxational and diffusive dynamics of T/G mixtures by complementary neutron scattering techniques [39,40,41,42]. 

**Figure 2:**
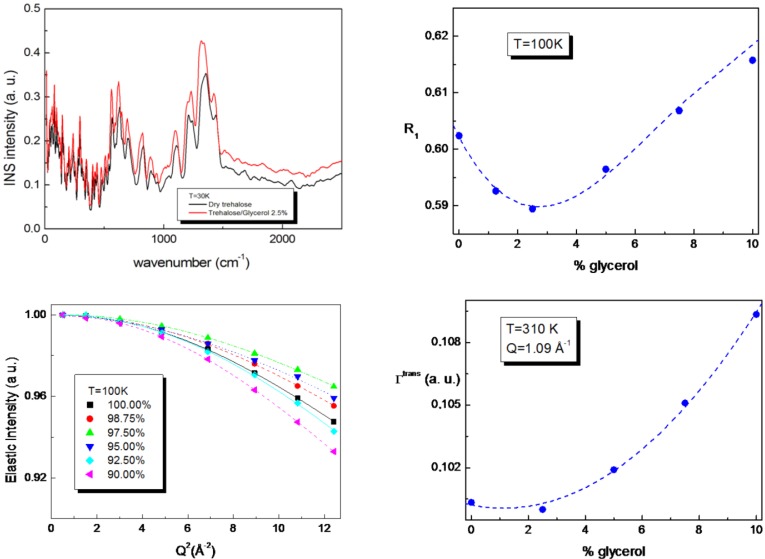
Vibrational, relaxational and diffusive properties of trehalose/glycerol mixtures [39,40,41,42].

The vibrational spectral region from 0 cm^−1^ to 2500 cm^−1^, shown in Figure 2, has been investigated by Inelastic Neutron Scattering [39] in order to understand the molecular mechanisms of the trehalose-glycerol interactions at different glycerol content. By the analysis of both the intramolecular and intermolecular motions of pure trehalose and of T/G mixtures, it emerges that at the glycerol content of 2.5%, the hydrogen bonded network strength of trehalose is mostly affected by the presence of glycerol, while a higher amount of glycerol does not have remarkable effects. 

Furthermore, the relaxational behavior of T/G mixtures revealed the presence of an excess vibrational contribution at low energy by varying concentration. The R_1_ fragility parameter has been evaluated in order to take into account the relative weight between the relaxational to vibrational contribution [40,41,42], showing that both at low and high temperature values, a minimum at the glycerol content of 2.5%, and then revealing a stronger character of the T/G mixtures at this concentration value. In addition, the decrease of the elastic intensity as a function of Q^2^ for the T/G mixture with a glycerol content of 2.5% (see Figure 2) is less marked than pure trehalose and the other mixtures, so confirming a higher rigidity at this glycerol content. Analogously, the derived mean square displacement behavior as a function of concentration for T/G mixtures shows a minimum at the concentration value of 2.5% by weight of glycerol.

Finally, Quasi Elastic Neutron Scattering allowed us to characterize the diffusive dynamics of T/G mixtures by evaluating the translational line width behavior as a function of Q and by extracting the diffusion coefficient values. The results show that the anomaly in the dynamics observed at low frequency is still present, since a minimum in the translational line width behavior occurs at the glycerol concentration value of 2.5% (Figure 2), so revealing a slowing down of the diffusive dynamics at this concentration value. These findings show that both the local and diffusive dynamics, which are linked to the stabilizing action on biomolecules, are suppressed at a very low glycerol concentration value, suggesting that for this glycerol content, the atomic attractive interactions are the strongest among the investigated concentration values. 

### 2.3. Interaction Mechanisms between Bioprotectant Systems and Biomolecules

Let us present the results obtained by neutron scattering techniques on lysozyme/trehalose/water mixtures and dUTPase/trehalose/water mixtures [43,44,45,46]. 

**Figure 3:**
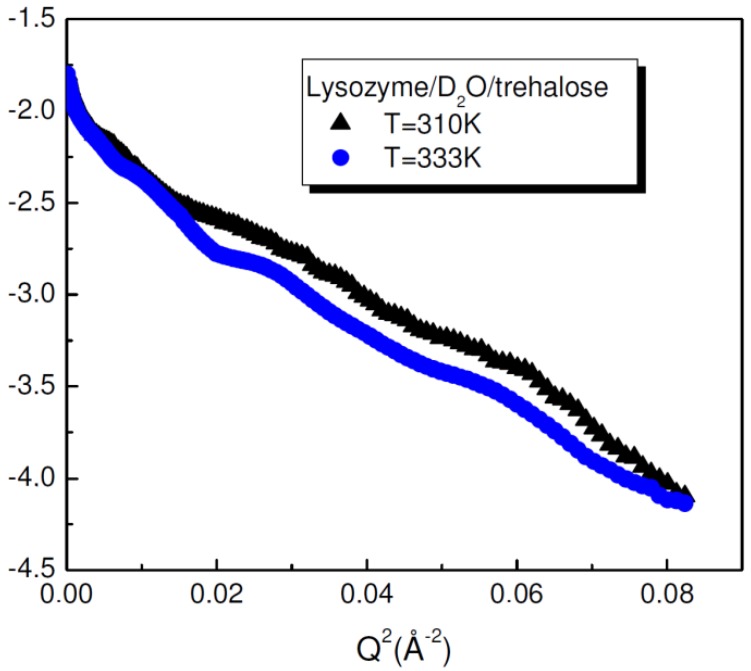
Guinier plot of the intensity profiles of lysozyme/D_2_O/trehalose mixtures as a function of *Q* for temperature values of T = 310 K and T = 333 K [43].

The conformational properties of lysozyme in the presence of trehalose as a function of temperature have been investigated by Small Angle Neutron Scattering in order to determine the conformational properties of the protein in presence of the disaccharide. By considering the Guinier relation [45,46], the size of the protein can be evaluated by allowing us to extract the gyration radius of lysozyme/D_2_O/trehalose solutions at different temperature values. The R_g_ values remain almost constant (16.2 Å at T = 310K and R_g_ = 16.4 Å at T = 333K) even when temperature increases, this thermal change inducing in absence of trehalose a conformational change in lysozyme (Figure 3). This result emphases the stabilizing effect of trehalose on lysozyme and the capability of trehalose to significantly inhibit the swelling of lysozyme induced by thermal stress.

**Figure 4:**
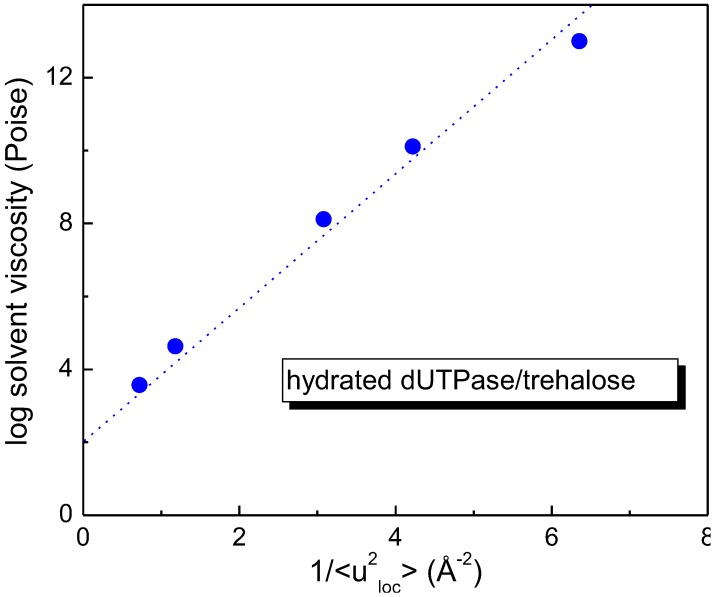
Linear dependence of viscosity of the trehalose/H_2_O mixture on the local mean square displacement of the hydrated dUTPase/trehalose system [44,45,46].

On the other hand, the dynamic properties of dUTPase protein immersed in a trehalose matrix have been investigated in order to study the effect of the host solvent on the protein dynamics. As shown in Figure 4, where the viscosity of the trehalose/H_2_O mixtures is plotted as a function of the local mean square displacement of the D_2_O-hydrated dUTPase/trehalose system, a linear relationship between the solvent, composed by trehalose and water, and the mean square displacement of hydrated dUTPase/trehalose system is verified. This result is a signature of a strong coupling between protein and the surrounding matrix, showing that a correlation exists between the protein dynamics and the viscosity of the surrounding environment [46]. 

## 3. Experimental Section

During the last year, several complementary techniques have been used to get the spectroscopic findings shown in the present review article. Here, we present an overview of the experimental details of the performed measurements.

Elastic Neutron Scattering measurements have been carried out across the glass transition temperature values by using the backscattering spectrometer IN13 at the Institute Laue Langevin (ILL, Grenoble, France). The IN13 main characteristics is the relatively high energy of the incident neutrons (16 meV), which makes it possible to span a wide range of momentum transfer Q (≤5.5 Å^−1^) with a very good energy resolution (~8 μeV). Therefore, neutron scattering experiments on IN13 provide information on the motions of the sample hydrogens in a space-time window of 1 Å and 0.1 ns given by its scattering vector modulus, Q, range and energy resolution, and allow us to characterize both flexibility (obtained from the fluctuation amplitudes) and rigidity (obtained from how fluctuations vary with temperature and expressed as a mean environmental force constant). 

Inelastic Neutron Scattering measurements have been performed by using the TOSCA indirect geometry time-of-flight spectrometer at the ISIS Pulse Neutron Facility (Rutherford Appleton Laboratory, Oxford, UK). The high energy resolution of TOSCA (Δ*E*/*E* ≈ 1.5%–2% for energy transfers up to several hundred meV) coupled with the high intensity of the ISIS source makes TOSCA ideal for studying the dynamics of water and aqueous mixtures below 2000 cm^−1^ (250 meV). TOSCA has revealed itself to be very effective in providing detailed results combined to optical spectroscopic techniques, such as Raman spectroscopy because of its design associating a single momentum transfer with each energy transfer. 

Quasi Elastic Neutron Scattering experiments were carried out by using the OSIRIS and IRIS spectrometers at the ISIS Facility (Rutherford Appleton Laboratory, Oxford, UK) and by using the IN4 and IN6 spectrometers at the Institute Laue Langevin (ILL, Grenoble, France). OSIRIS, situated on the N6(B) beam line at ISIS, is an inverted geometry time-of-flight instrument such that neutrons scattered by the sample are energy analyzed by means of Bragg scattering from large-area crystal-analyzer array. It can be used as either a high-resolution, long-wavelength diffractometer or for high-resolution quasi/inelastic neutron scattering spectroscopy. The configuration of OSIRIS used for the INS measurements was: scattering angle range of 11° < 2θ < 55°, PG004 graphite with a momentum transfer range of 0.7Å^−1^ < Q < 3.6 Å^−1^ and energy resolution of 99 μeV (FWHM). IRIS, which is also an inverted geometry spectrometer, has been used in the high resolution configuration, *i.e.*, graphite 002 and mica 006 analyzer reflections, to measure sets of QENS spectra covering a Q,ω-domain extending from hω = −0.3 to 0.6 meV and Q = 0.5 to 1.8 Å^−1^. The used detectors give a mean energy resolution of Γ = 8 μeV (HWHM) as determined by reference to a standard vanadium plate. The IN4 spectrometer is a time-of-flight spectrometer used for the study of excitations in condensed matter, and it was configured for the measurements with an incident wavelength of 2.96 Å and an energy resolution of 450 μeV. The IN6 spectrometer is a time-of-flight spectrometer designed for quasi-elastic and inelastic scattering for incident wavelengths in the range of 4 to 6 Å. The incident wavelength used for the measurements was 5.12 Å with an energy resolution of 50 μeV. 

Small Angle Neutron Scattering experiments have been performed by using the LOQ spectrometer at the ISIS Facility (Rutherford Appleton Laboratory, Oxford, UK) for different contrast values (20 H_2_O-80% D_2_O, 80 H_2_O-20% D_2_O and 100% D_2_O). The contrast variation technique collecting data at different D_2_O/H_2_O molar ratio has been employed in order to determine the protein scattering density length. The Q-range covered by the LOQ spectrometer in this experiment is from 0.007 Å^−1^ to 0.287 Å^−1^. Incoming neutrons are monochromatized by a mechanical velocity selector with variable wavelength from 2.2 to 10.0 Å, the wavelength resolution (FWHM) being 8% < Δλ/λ < 18%.

## 4. Conclusions

All the studies performed on trehalose water mixtures clearly support the hypothesis of a privileged water-disaccharide interaction. Both the results dealing with structural and dynamic properties suggest that on the one hand, trehalose binds more strongly to water molecules, so disrupting their tetrahedral configuration arrangements and slowing down their mobility, and on the other hand, trehalose shows a larger structural resistance to temperature changes and a higher “rigidity” in comparison with its homologues. 

The physical picture obtained from the studies performed on trehalose water mixtures shows that the higher bioprotectant effectiveness of trehalose in comparison with the other disaccharides is due to the combined effect of different co-factors. What emerges is that trehalose, besides modifying significantly the structural and dynamical properties of water, forms with H_2_O a less fragile entity able to encapsulate biological structures and to protect them in a more rigid environment. Due to the fundamental role of water in living organisms as its major component and as the prerequisite for proteins and cells to exert their biological functions, the elucidation of the bioprotectant-water interactions can explain the bioprotective functions under the harsh conditions encountered in extreme environment.

The whole body of data on trehalose/glycerol mixtures at different glycerol content support the hypothesis that in this small glycerol concentration range, the T/G matrix forms a stronger hydrogen bonding network with respect to that of pure trehalose and to what happens for higher glycerol concentration values. The signature of a strengthening of the hydrogen bonded network created by trehalose and glycerol is recognizable in the trends followed by all the determined physical quantities. More specifically, the increased rigidity revealed by the dynamic features confirms that the hydrogen bonded interactions are rearranged in a stronger network as a consequence of the addition of glycerol. The molecular origin of this anomalous behavior can be linked to the registered minima in the mean square displacement, in the *R_1_* parameter and in the translational line width, which clearly signals the presence of a not-ideal mixing process. 

Furthermore, it is to be observed that the inelastic data were collected at very low temperature, therefore, they can have interesting implications about the described combined role of the trehalose/glycerol system as a cryo- and lyo-protectant system. The occurrence of large amounts of trehalose and small amounts of glycerol in several organisms capable of activating a cryoprotective dehydration process can find physical elucidation in the present findings. 

It is known that the coupling between the dynamics of the host medium with that of the protein may explain the bioprotectant function. The results on lysozyme and dUTPase in the presence of trehalose emphasize that proteins are complex systems, which are to be considered as dynamic systems that perform motions to execute their functions. These motions actually involve the atoms not just of the biological structure itself, but also of the surrounding medium with which a coupling exists. Therefore, depending on the circumstances, the protein can be considered “slaved” or “sequestered” by the host medium, which may be able to suppress its dynamics, so resulting in a retardation of denaturation processes or a slowing down of biological function, as happens in extreme conditions.

The findings on the binary bioprotectant mixtures and on the ternary bioprotectant/biomolecule systems provide precious information to explain at a molecular level the behavior of biomolecules under stress conditions. Here, since all the shown data have been collected as a function of temperature in a wide range (20 K–400 K), thermal stress plays a key role. 

The neutron scattering data at very high temperatures can help in the understanding of the dynamic nature of hyperthermostable proteins due to the unraveling of the mechanism responsible for the balance between rigidity, which is related to heat resistance, and molecular fluctuations at high temperatures, which account for biological function. On the other hand, the findings at very low temperatures can support the hypothesis that cold-temperature adapted proteins from psychrophiles become more rigid, implying that enhancing flexibility can restore function. Oother useful suggestions are furnished by the data on trehalose/glycerol mixtures based on the elucidation of their interaction, this circumstance being crucial for halophile organisms.
